# Positive Youth Development: Parental Warmth, Values, and Prosocial Behavior in 11 Cultural Groups

**DOI:** 10.5195/jyd.2021.1026

**Published:** 2021

**Authors:** Concetta Pastorelli, Antonio Zuffianò, Jennifer E. Lansford, Eriona Thartori, Marc H. Bornstein, Lei Chang, Kirby Deater-Deckard, Laura Di Giunta, Kenneth A. Dodge, Sevtap Gurdal, Qin Liu, Qian Long, Paul Oburu, Ann T. Skinner, Emma Sorbring, Laurence Steinberg, Sombat Tapanya, Liliana Maria Uribe Tirado, Saengduean Yotanyamaneewong, Suha Al-Hassan, Liane Peña Alampay, Dario Bacchini

**Affiliations:** 1 Sapienza University of Rome;; 2 Duke University;; 3 Eunice Kennedy Shriver National Institute of Child Health and Human Development;; 4 University of Macau;; 5 University of Massachusetts, Amherst;; 6 University West, Trollhättan;; 7 Chongqing Medical University;; 8 Duke Kunshan University;; 9 Maseno University;; 10 Temple University and King Abdulaziz University;; 11 Chiang Mai University;; 12 Universidad de San Buenaventura, Medellin;; 13 Hashemite University, Jordan and Emirates College for Advanced Education, Abu Dhabi;; 14 Ateneo de Manila University;; 15 University of Naples “Federico II”

**Keywords:** parental warmth, family values, prosocial behavior, cross-cultural, positive youth development, adolescence

## Abstract

The current cross-cultural study aimed to extend research on parenting and children’s prosocial behavior by examining relations among parental warmth, values related to family obligations (i.e., children’s support to and respect for their parents, siblings, and extended family), and prosocial behavior during the transition to adolescence (from ages 9 to 12). Mothers, fathers, and their children (N = 1107 families) from 8 countries including 11 cultural groups (Colombia; Rome and Naples, Italy; Jordan; Kenya; the Philippines; Sweden; Thailand; and African Americans, European Americans, and Latin Americans in the United States) provided data over 3 years in 3 waves (M_age_ of child in wave 1 = 9.34 years, SD = 0.75; 50.5% female). Overall, across all 11 cultural groups, multivariate change score analysis revealed positive associations among the change rates of parental warmth, values related to family obligations, and prosocial behavior during late childhood (from age 9 to 10) and early-adolescence (from age 10 to 12). In most cultural groups, more parental warmth at ages 9 and 10 predicted steeper mean-level increases in prosocial behavior in subsequent years. The findings highlight the prominent role of positive family context, characterized by warm relationships and shared prosocial values, in fostering children’s positive development in the transition to adolescence. The practical implications of these findings are discussed.

## Introduction

Prosocial behaviors, defined as voluntary actions aimed to benefit others, are important for adaptation and health throughout the life course (Caprara et al., 2000; Eisenberg & Fabes, 1998; Hoffman, 2000). A large body of research has shown that being prosocial is associated with greater agreeableness, openness, and self-esteem (Luengo Kanacri et al., 2014; Zuffianò et al., 2014); academic and career achievement (Caprara et al., 2000); optimal relational skills (Eisenberg et al., 2015), and other oriented values that assign priority to the welfare of others (both in-group and out-group members; Schwartz, 2010). Furthermore, the beneficial effects of prosocial behavior through constructive and mutually rewarding interpersonal relationships protect against the risks of maladjustment (e.g., Kokko et al., 2006).

Caring and character development are core components of positive youth development (PYD), and the study of prosocial behaviors lies clearly within the PYD framework (Damon, 2004). Since the introduction of influential models of PYD (Lerner et al., 2005), researchers have increased their endeavors to study the positive development of children and adolescents to identify factors that promote youth thriving and well-being. The focus of developmental science on plasticity and strengths in human behavior rapidly expanded and counterbalanced the deficit model that had been pervasive in much of psychology (see Lerner at al., 2015). According to the PYD framework, studying strengths and assets available in home, school, and community contexts advances understanding of bidirectional relations between youth and their contexts over time, as well as how to promote positive youth development within these contexts (Lerner, 2006; Lerner et al., 2015).

Following this line of research, in the present study, we aimed to advance knowledge on the dynamic transactions between parenting and children’s prosocial behaviors. In particular, we examined prosocial development in relation to familial and contextual influences and changes during the transition to adolescence, using two distinctive dimensions of parenting, namely parental warmth and values about expectations for family obligations (e.g., respect for elder members, time spent with family). We used three waves of data collected from 11 cultural groups across the globe when children were ages 9 to 12 years. We first outline the developmental foundations of prosocial behavior during adolescence and then link its development to the role played by family characteristics (i.e., parental warmth and family values).

### Adolescence and Prosocial Behavior: A Developmental Analysis

The transition to adolescence is characterized by multiple biological, cognitive, and social changes. Most adolescents perceive and internalize social norms and related values that derive from their proximal family context, while simultaneously being exposed to other influences outside the family (Hart & Carlo, 2005). As adolescents start to navigate their expanded social world, socio-cognitive and moral development help adolescents recognize their proactive role in the family and larger society. Adolescents become more cognizant of opportunities to behave prosocially outside their home (Eisenberg et al., 2015). Parents adjust their behavior to accommodate the needs of their growing children, providing forms of guidance that grant their children greater independence while still supporting them in navigating the challenges of the social world (Smetana & Rote, 2019). All of these changes have an impact on the functioning of the family system, and this developmental transition represents an important window for examining the dynamic relations between parenting and prosocial behavior.

Developmental findings on prosocial behavior also highlighted its malleable nature and how socialization contexts can contribute to its change and continuity (e.g., Bandura 1977; Knafo & Plomin, 2006). Ample evidence suggests that children’s prosocial development is nurtured through positive experiences in close relationships within the family (see Eisenberg et al., 2015). Caregivers provide learning experiences that may enhance children’s prosocial development through their prosocial behavior (model) and expectations (cognitions) about valued actions that benefit others. Observing and being prompted to engage in other-oriented actions also stimulate youth to become aware of the importance of their contributions to the social group and their acquisition of a social identity (Miller & Goodnow, 1995).

Despite notable advances in understanding prosocial development, studies have been conducted primarily in Western, educated, industrialized, rich, and democratic societies (so-called WEIRD; Henrich et al., 2010). Hence, the extent to which cultural differences may account for the development of prosocial behavior remains partly unexplored. Accordingly, in the present study, we examined the longitudinal relations among parental warmth, values related to expectations for family obligations, and prosocial behavior in 11 cultural groups. Understanding the relations among these three developmental processes may help identify the extent to which these constructs develop simultaneously, whether development in one factor shapes subsequent changes in the other factors, and whether bidirectional relations characterize the links among the factors.

### Parental Warmth and Adolescents’ Prosocial Behaviors

Although specific aspects of parenting (e.g., inductive discipline) likely affect prosocial behavior (Krevans & Gibbs, 1996), a consistent body of empirical research has highlighted the role of parental warmth as a key factor for children’s prosocial actions (Carlo et al., 2010; Laible et al., 2000; Padilla-Walker et al., 2012; Pastorelli et al., 2016; Putnick et al., 2015, 2018; Zhou et al., 2002). At least three theoretical explanations account for this link. First, parental warmth may enhance children’s prosocial skills through modeling (Bandura, 1977). That is, a caring parent offers a model of empathic, helping, and comforting behaviors that children may emulate (Bandura, 1977). Second, parental actions that offer children feelings of trust and protection may enhance children’s sense of connection to and concern for others (Eisenberg et al., 2015; Hoffman, 2008). Third, a warm parent–child relationship creates rewarding affective ties that are conducive to optimal emotional self-regulation, empathy, and prosocial actions. An extensive body of literature has examined the relation between parental warmth and children’s prosocial behavior in early and late childhood (for a review see Eisenberg et al., 2015). Yet, research focusing on the relation between parental warmth and adolescents’ prosocial behavior is still scarce, leaving open the possibility of developmental (dis)continuity in their positive relation from childhood to adolescence.

Most studies exploring developmental transactions show positive reciprocal associations between parental warmth and children’s prosocial behavior over time. For example, in a longitudinal study examining reciprocal relations between authoritative parenting (i.e., a combination of high parental connection, regulation, and autonomy granting) and adolescents’ prosocial behavior toward family members from ages 12 to 13, Padilla-Walker et al. (2012) found that adolescents’ prosocial behavior at age 12 positively predicted authoritative mothering and fathering a year later, and authoritative mothering at age 12 positively predicted adolescents’ prosocial behavior a year later. Similarly, Carlo et al. (2018) showed that authoritative parents (high levels of warmth and low levels of harshness) were more likely to have children (ranging in age from 10 to 14) who exhibited high levels of prosocial behaviors across time than parents who were moderately demanding and less involved. In another study, Carlo et al. (2010) also found bidirectional relations between maternal warmth and adolescents’ prosocial behavior during the transition to adolescence.

Finally, in previous studies using the same longitudinal data set of the current study, bivariate relations were tested using traditional cross-lagged models and provided information on the links between prosocial behavior and parenting variables related to warmth and involvement or to parental acceptance in different countries. In Pastorelli et al. (2016), covering two time points, mother and child reports of prosocial behavior at age 9 contributed to child report of the quality of mother–child relationships (high parental warmth and involvement) at age 10, but not the reverse. In Putnick et al. (2018), covering three data points from age 9 to 12, reciprocal relations between the overall construct of parental acceptance (mother and father report) and self-reported child prosocial behavior were found during middle childhood, but not in early adolescence. However, the present study is the first to adopt a multivariate framework to study the parallel development (i.e., the extent to which these constructs develop in a parallel fashion) and predictive relations between the specific dimension of parental warmth and prosocial behaviors. The present study also extends the investigation of links between parenting and children’s prosocial behavior to encompass values related to expectations for family obligations and to examine their development over time.

### Parental Warmth and Values and Child Prosocial Behavior

In addition to parental warmth, parental values constitute essential elements in the parent-child socialization process (Bornstein, 2015). Values are goals and guiding principles in people’s behavior (Schwartz, 1992). Parental goals motivate and guide the quality of parents’ relationships with their children, their practices, and their expectations about children’s positive behaviors (Darling & Steinberg, 1993). High parental warmth creates a positive climate conducive to children’s willingness to accept parental values related to the respect and care of others. The transmission of parents’ values to children follows a sequence in which children first must perceive parents’ values and then either endorse or reject those parental values (Grusec & Goodnow, 1994). The process of internalization of parents’ values largely depends on children’s age and the quality of parent–child relationships, as well as on other relationships within and outside the family (peers; Trommsdorff, 1995). Parents can foster the internalization of children’s values of caring and respect by acting as role models and stressing the importance of closeness and support among family members. For instance, parents who facilitate children’s disclosure of their thoughts and feelings, treat them kindly and gently, and make them feel loved and important contribute to a warm climate within the family that is conducive to children’s willingness to internalize parental values. The construct of family obligations (i.e., parental expectations that children should respect, assist, and support family members; Fuligni et al., 1999) plays a major role due to its relevance for children’s prosocial behavior across different cultures (e.g., Colombia, the Philippines, Kenya, and Italy; see Lansford et al., 2018). A family environment in which the values of support and respect are salient is optimal for children’s prosocial development (Fuligni, 2019).

According to Fuligni and colleagues’ model (1999; Fuligni, 2007), expectations for family obligations consist of two dimensions: family respect and assistance. Family respect concerns the importance parents assign to older family members (e.g., parents, grandparents, and older siblings) and sacrifices children should make for their family. Family assistance reflects parents’ expectations that their children will help family members (e.g., run errands for the family, help around the house). Because this model has been tested in different cultural groups and provides a clear framework for understanding family obligations, in our study we considered both parental expectations and children’s perceptions of their parents’ expectations regarding family obligations to obtain a more comprehensive perspective on family values. A previous study conducted by our research group examining expectations for family obligations in 13 cultural groups found that more parental warmth and less hostility, rejection, and neglect at age 8 predicted later family obligations at age 10, confirming that parents’ and children’s expectations for family obligations are facilitated by a warm, supportive family climate (Lansford et al., 2018).

Other studies also demonstrate relations among positive parenting, family-related values, and children’s prosocial behaviors. For example, in a cross-sectional study with a sample of Mexican American adolescents (*M*_*age*_ = 10.91 years) and their mothers, Calderón et al. (2011) found that mothers’ familism values related to the importance of family support and obligations to help and share resources with relatives were associated with inductive parenting and children’s prosocial behaviors within and outside of the family context, which in turn were related to adolescents’ perceptions of inductive parenting. Furthermore, adolescents’ endorsement of familism values partially mediated the relation between adolescents’ perceptions of prosocial parenting and prosocial behaviors. Padilla-Walker and Carlo (2007) found that maternal expectations for prosocial behaviors were linked to adolescents’ prosocial behavior through adolescents’ endorsement of prosocial values. In other words, adolescents’ prosocial values mediated the relation between adolescents’ perceptions of maternal expectations and adolescents’ prosocial behaviors.

The relation between familism values and prosocial behavior during adolescence is also supported by some longitudinal studies. For example, in a longitudinal study with a sample of 749 Mexican American adolescents and their parents, Knight et al. (2016) found that parents’ familism values when children were approximately 10 years old predicted adolescents’ familism values two years later, which, in turn, positively predicted several types of adolescents’ prosocial behaviors three years later. In addition, Knight et al. (2018) found that those adolescents who were higher in familism values in fifth grade and decreased less in their familism values from fifth to 10^th^ grade reported more prosocial tendencies at 12^th^ grade, after controlling for 10^th^-grade levels of these prosocial tendencies.

Hence, there is some evidence that children’s perceptions of their parents’ values and expectations are associated with children’s prosocial behavior. Yet, the extent to which the strength of these positive relations is stable across both Western and non-Western samples deserves further work to ascertain its universality or culture-specificity.

### The Current Study

Considering the scarcity of studies that have examined the relations among positive parenting, family values, and children’s prosocial behavior during the transition to adolescence (e.g., Calderón et al., 2011), the present study aimed to advance understanding of the longitudinal associations among changes in parental warmth, values related to family obligations, and children’s prosocial behavior in a set of diverse countries around the world during the transition to adolescence. In particular, whereas previous longitudinal work used classic statistical models that mostly captured interindividual differences (e.g., cross-lagged model; Padilla-Walker et al., 2012; Pastorelli et al., 2016; Putnick et al., 2018), in the present study we used a multivariate change score model (McArdle, 2009), which allowed us to simultaneously consider interindividual variability in intra-individual development. As suggested by Grimm (2007), “development is a complex, multifaceted, multidimensional, and multidirectional process with multiple causes and consequences” (p. 338) and understanding the extent to which these constructs develop simultaneously and whether one developmental process could predict another one is of great importance to advance theoretical knowledge as well as to inform appropriate interventions.

In summary, we examined two research questions. First, to what extent did changes in parental warmth and values with children’s prosocial behaviors jointly develop over time in 11 cultural groups? We hypothesized that steeper mean-value increases in one construct (e.g., warmth) would be related to parallel mean-value increases in another construct (e.g., prosocial behavior) in the same period (i.e., from age 9 to 10; for a similar approach see Quin et al., 2020). Although the importance of respect and care for family members as well as warmth and affection in parent–child relationships are key constituents of children’s socialization and prosocial development across societies (Bornstein et al., 2012), the strength of these associations might vary across cultural groups. Second, following Grimm et al. (2012), we also investigated whether previous values (e.g., age 10) and prior changes (e.g., from age 9 to age 10) in one construct predicted subsequent changes (e.g., from age 10 to age 12) in another construct (e.g., prosocial behavior). Consistent with previous studies (e.g., Padilla-Walker et al., 2012; Putnick et al., 2018), we expected positive cross-construct predictive effects (both in terms of initial level and changes) of parental warmth and family values on later child prosocial behavior. We recognize that the strengths of these effects could vary across cultural groups.

## Method

### Participants

Families (*N* = 1107) including 1107 mothers, 975 fathers, and 1107 children (50.5% female) from 8 countries provided data over 3 years in three waves. Children were approximately 9 years old (*M*_*age*_ = 9.34, *SD* = 0.75; Time 1[T1]), 10 years old (*M*_*age*_ = 10.38, *SD* = 0.74; Time 2 [T2]), and 12 years old (*M*_*age*_ = 12.90, *SD* = 0.81; Time 3 [T3]). Participating families were from 11 cultural groups, namely Medellin, Colombia (*n*s = 101 children, 101 mothers, and 100 fathers); Naples, Italy (*n*s = 99 children, 99 mothers, and 95 fathers); Rome, Italy (*n*s = 101 children, 101 mothers, and 95 fathers); Zarqa, Jordan (*n*s = 114 children, 113 mothers, and 111 fathers); Kisumu, Kenya (*n*s = 95 children, 95 mothers, and 92 fathers); Manila, Philippines (*n*s = 107 children, 107 mothers, and 94 fathers); Trollhättan/Vänersborg, Sweden (*n*s = 97 children, 99 mothers, and 83 fathers); Chiang Mai, Thailand (*n*s = 116 children, 116 mothers, and 99 fathers); and Durham, North Carolina, United States (*n*s =100, 91, and 85 children, *n*s =100, 91, and 85 mothers, and *n*s = 89, 45, and 69 fathers, respectively for European Americans, African Americans, and Latin Americans). At T1, mothers averaged 38.30 years of age (*SD* = 6.68), and fathers averaged 41.47 years of age (*SD* = 6.89). Mothers had completed 12.59 years of education (*SD* = 4.29), and fathers had completed 12.87 years of education (*SD* = 4.18) on average. Mothers reported that 80% were married, 7.20% were cohabitating, and 5.9% were unpartnered. Nearly all were biological parents, with 4% being grandparents, stepparents, or other adult caregivers. Supplementary Table S1 displays sociodemographic characteristics of the sample by country.

Sampling focused on including families from the majority ethnic group in each country; two exceptions were in Kenya where we sampled Luo (3^rd^ largest ethnic group, 13% of the population) and in the United States where we sampled equal proportions of European American, African American, and Latino families. The participating countries were selected because they vary on several sociodemographic dimensions, including predominant ethnicity and religion, economic indicators, and indices of child well-being, and on psychological constructs, such as individualism/collectivism, which provides a sample of countries that better represent the diversity of youths’ experiences around the world than has been the case in much prior research. For example, regarding the sociodemographic dimensions, on the Human Development Index, a composite indicator of a country’s status with respect to health, education, and income, participating countries had ranks of 8 for Sweden, 15 for United States, 29 for Italy, 77 for Thailand, 79 for Colombia, 102 for Jordan, 106 for the Philippines, and 147 for Kenya, out of 189 countries with available data, indicating that the countries in the sample ranked from among the highest to among the lowest on the composite indicator of factors related to human development (United Nations Development Program, 2019).

Retention rates were high during the longitudinal data collection of the present study: 92% of the original sample continued to provide data 1 year after Wave 1 of the present study; 77% of the original sample continued to provide data 3 years after Wave 1 of the present study. The attrition was mainly due to the unavailability of individuals to take part in the later phases of the study or our inability to contact the participant. Attrited participants did not demographically differ from the original sample with respect to child gender, parents’ age and education, or family income.

### Procedures

Families were recruited from schools that served socioeconomically diverse populations in each participating country. Recruitment letters describing the larger Parenting Across Cultures study were sent home with children, and parents were asked to return a signed form if they were willing to be contacted about the study. Families were then enrolled in the study until the target sample size (approximately 100 families) was reached in each country. To guarantee coverage of families from different socioeconomic backgrounds, students from private and public schools were sampled in approximate proportions to which they were represented in the local population.

A procedure of forward- and back-translation was used to ensure the linguistic and conceptual equivalence of measures across languages (Erkut, 2010). Translators were fluent in English and the target language. In addition to translating the measures, translators were asked to note and suggest improvements to items that did not translate well, were inappropriate for the participants, were culturally insensitive, or elicited multiple meanings. Site coordinators and the translators reviewed the discrepant items and made appropriate modifications. Measures were administered in Spanish (Colombia and the United States), Italian (Italy), Arabic (Jordan), Dholuo (Kenya), Filipino (the Philippines), Swedish (Sweden), Thai (Thailand), and American English (the United States and the Philippines).

Interviews lasted 1.5 to 2 hours and were conducted in participants’ homes, schools, or at other locations chosen by the participants. Procedures were approved by local Institutional Review Boards (IRBs) at universities in each participating country; mothers and fathers provided written consent, and children provided assent, and all were interviewed separately to ensure privacy. Parents were given the option of having the questionnaires administered orally (with rating scales provided as visual aids) or completing written questionnaires. Children completed the measures orally. Depending on the site, families were given modest financial compensation for their participation, families were entered into drawings for prizes, or modest financial contributions were made to children’s schools.

### Measures

Participants’ parental warmth, family values, and prosocial behaviors were each assessed with a multi-informant approach. Specifically, parental warmth and family values combined child, mother, and father reports, and children’s prosocial behaviors combined mother and father reports. At each time point, we averaged scores across all available informants to create an overall score for each construct. Cross-informant, within-time correlations ranged from .28 to .42 (parental warmth), from .32 to .54 (family values), and from .38 to .46 (prosocial behaviors). Approximate measurement invariance was demonstrated for all constructs using the alignment method (Muthén & Asparouhov, 2014; see Supplementary Materials for details on the invariance tests).

#### Parental Warmth

Children, mothers, and fathers independently completed the Parental Acceptance–Rejection/Control Questionnaire-Short Form (PARQ/Control-SF; Rohner, 2005) to measure the frequency of parenting behaviors (Rohner, 2005). In the present study, we focused on the eight-item warmth–affection subscale. Children and parents rated the frequency of each behavior (e.g., parent version “I make my child feel wanted and needed”; child version “My mother/father makes me feel wanted and needed”) on a modified 4-point scale: 1 (*never or almost never*), 2 (*once a month*), 3(*once a week*), or 4 (*every day*). Children completed the measure twice, once for each parent. Reliability coefficients (α) across countries were: .77, .70, and .69 for child-perceived maternal warmth; .82, .84, and .86 for child-perceived paternal warmth; .78, .79, and .75 for mother self-report of warmth; and .80, .82, and .79 for father self-report of warmth at the three time points, respectively.

#### Family Values

Children, mothers, and fathers completed the Respect for Family and Current Assistance scales of the Family Obligations Measure developed by Fuligni et al. (1999). The measure includes seven items assessing views about the importance of respecting the authority of elders in the family, including parents, grandparents, and older siblings on a 5-point scale from 1 (*not important*) to 5 (*very important*) and 11 items assessing parents’ expectations and children’s perceptions of their parents’ expectations regarding how often children should help and spend time with the family on a daily basis on a 5-point scale from 1 (*almost never*) to 5 (*almost always*). Data for family values were collected starting from T2. Reliability coefficients (α) across countries were .83 and .85 for child self-report of family values, .84 and .84 for mother self-report of family values, and .86 and .87 for father self-report of family values at T2 and T3, respectively.

#### Prosocial Behaviors

Mothers and fathers rated their children’s tendency to behave prosocially using three items from the Children’s Prosociality Scale (Caprara & Pastorelli, 1993; Pastorelli et al., 1997). Parents rated each item (e.g., “He/she tries to help others”) using a 5-point rating scale from 1 (*never*) to 5 (*often*). The validity and reliability of the Prosociality Scale have been cross-nationally demonstrated on large samples (Caprara et al., 2005; Pastorelli et al., 1997). Reliability coefficients (α) across countries were .63, .64, and .65 for mother reports, and .58, .66, and .70 for father reports at the three time points, respectively.

#### Socioeconomic Status (SES)

In line with previous work indicating parents’ educational level as a key indicator of family SES (e.g., Mistry et al., 2002), we used an average of the years of education reported by both mothers and fathers as a proxy for SES.

### Data Analytic Approach

**Figure 1.**
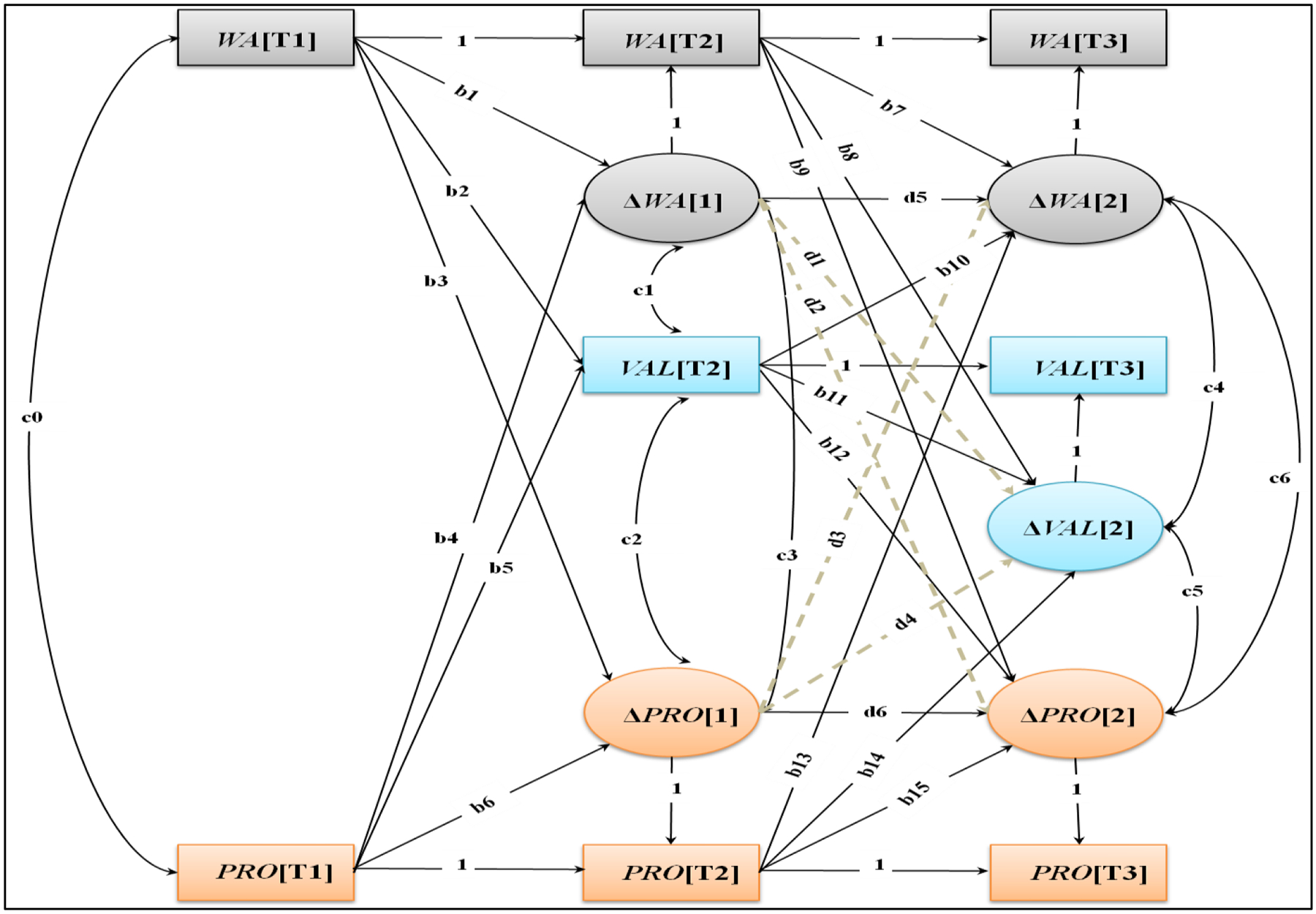
Multivariate Change Model of Parental Warmth, Family Values, and Children’s Prosocial Behavior. *Note*. Multivariate change model of parental warmth (WA), family values (VAL), and children’s prosocial behavior (PRO) at Time 1 (T1 = 9 years of age), Time 2 (T2 = 10 years of age), and Time 3 (T3 = 12 years of age). Change factors (Δ) in the first transition (i.e., from T1 to T2; [1]) and second transition (i.e., from T2 to T3; [2]) are reported in circles. Parameters with a label (e.g., “c0”) were freely estimated.

To examine the simultaneous developmental processes among parental warmth, values, and children’s prosocial behaviors, we used a multivariate change scores analysis (McArdle, 2009) in each cultural site. Given the complexity of our analytic approach and to ease model convergence, we worked at the observed level (i.e., the constructs and the change factors were not estimated at the latent level; for a similar specification, see Valente & MacKinnon, 2017). As shown in Figure 1, this approach allowed us to simultaneously consider: (a) relations between each change factor and prior levels in the constructs of interest (e.g., were mean-level changes in values from T2 to T3 predicted by previous levels of warmth, values, and prosocial behaviors at T2?), (b) predictive relations between change factors (did greater changes in warmth from T1 to T2 predict subsequent increases in prosocial behavior from T2 to T3?), and (c) the covariation among the change factors (e.g., were greater mean-level increases in parental warmth from T2 to T3 related to steeper increases in values and prosocial behaviors during the same transition?). To identify possible differences in the paths of interest across the 11 cultural sites, we used a multiple group framework in which the fit of the *unconstrained* model (i.e., all paths were freely estimated across the cultural groups) was compared to the fit of the *constrained* model (i.e., all paths were constrained to be equal across the cultural groups) via the delta chi-square test for nested models (Δχ^2^). Finally, we also checked the robustness of the results by including children’s gender and SES in our analyses. Missing data were handled with full information maximum likelihood (FIML; Arbuckle, 1996). To evaluate model fit, in addition to the χ^2^ test, we also considered the following indexes with their relative cut-off as a rule-of-thumb for acceptable fit: a comparative fit index (CFI) and Tucker-Lewis index (TLI) > .90 and a root mean square error of approximation (RMSEA) < .08 with 90% confidence intervals (CI). All models were run in M*plus* 8 (Muthén & Muthén, 1998–2017) with maximum likelihood with standard errors robust to nonnormality as a method of estimation.

## Results

### Preliminary Analyses

Means and standard deviations for the multi-informant constructs are presented in Supplementary Table S2. Multilevel modeling was used to examine the developmental trajectories of the constructs of interest. The results are reported in Supplementary Materials.

### Multiple-group Multivariate Change Analysis

We followed a three-step model-building procedure to analyze the joint developmental relations among parental warmth and family values and children’s prosocial behaviors. First, to achieve model over-identification (i.e., degrees of freedom greater than zero), we estimated a multiple-group model in which the cross-construct, change-to-change predictive effects were initially constrained to be zero (i.e., the grey paths in Figure 1). This model (Model 1 *unconstrained*) showed an acceptable fit, χ^2^ (44) = 64.362, *p* = .024, CFI = .987, TLI = .915, RMSEA = .073, 90% CI [.027, .109], but was statistically different, Δχ^2^(239) = 423.095, *p* < .001, from the constrained model (Model 1 *constrained*), χ^2^ (283) = 488.271, *p* < .001, CFI = .872, TLI = .866, RMSEA = .091, 90% CI [.077, .105], suggesting the presence of cultural specificities in the parameters estimated. After inspecting both the modification indexes and the chi-square contribution from each group, we relaxed some parameters in Kenya, Thailand, U.S. African Americans, and Jordan. This refined model (Model 1 *partially constrained*) had an acceptable fit, χ^2^ (262) = 305.709, *p* = .033, CFI = .973, TLI = .969, RMSEA = .044, 90% CI [.014, .063], and was not statistically different from the unconstrained model, Δχ^2^(218) = 242.522, *p* = .122. In this model we found that: (a) higher values of warmth at T1 significantly predicted (β ≈ .17, *p*s < .001) steeper increases in the change factor of prosocial behaviors from T1 to T2 in all cultural groups (path b3 in Figure 1) except for Kenya (β = .061, *p* = .542), Thailand (β = −.095, *p* = .292), and Jordan (β = −.017, *p* = .841), whereas higher values of warmth at T2 did not significantly (β ≈ .07, ps ranging from .064 to .078) predict higher mean-level changes in prosocial behavior from T2 to T3; (b) the correlations among the change parameters (parameters labeled c1–c6 in Figure 1) were always positive and statistically significant, indicating a consistent positive parallel development among parental warmth, family values, and prosocial behavior across countries (*r*s ranged from .041, *p* = .003, in Kenya [parameter c3 in Figure 1] to .571, *p* < .001 in Jordan [parameter c4 in Figure 1]). The positive joint development in the last transition (i.e., from T2 to T3) was significantly stronger in Jordan compared to other sites (see Table S3 in the Supplementary Materials).

Next, we used Model 1 (*partially constrained*) to add the cross-construct, change-to-change predictive effects. In this model (Model 2), χ^2^ (258) = 300.982, *p* = .034, CFI = .973, TLI = .969, RMSEA = .044, 90% CI [.013, .063], the cross-construct, change-to-change predictive effects were not statistically significant, and all were of similar size in each country (β ≈ −.05, *ps* >.075). Importantly, the addition of these further parameters did not alter the statistical significance of the positive joint development identified in the previous step.

Finally, in Model 3, we added the child gender and SES covariates to partial out their effects from the estimated parameters. Results from this model, χ^2^ (407) = 469.193, *p* = .018, CFI = .967, TLI = .961, RMSEA = .043, 90% CI [.019, .059], are reported in Table S2 in the Supplemental Materials. Overall, even while controlling for child gender (e.g., girls increased in their prosocial behavior from T1 to T2 more than boys) and SES (e.g., higher SES predicted lower mean-value changes in children’s prosocial behavior from T2 to T3), parental warmth and family values and children’s prosocial behaviors showed a consistent positive joint development across countries (which was stronger in Jordan from T2 to T3). In terms of predictive effects, higher values of warmth both at T1 (except for Kenya, Thailand, and Jordan) and T2 (all countries) predicted steeper mean-level increases in prosocial behavior in the subsequent transitions (i.e., from T1 to T2 and from T2 to T3, respectively). Final results from this model are reported in Table S3 in the Supplementary Materials.

## Discussion

Overall, across all 11 cultural groups, the findings of the present study revealed that changes in parental warmth, family-related values, and children’s prosocial behaviors jointly develop during late childhood and early adolescence, underlying the contribution of positive and interconnected family relationships to positive youth development. Caring relationships within the family are primary resources for positive youth development and assigning importance to values of respecting and helping other family members is likely to favor the development of a self-identity committed to moral-prosocial actions (Blasi, 1983).

Regarding the first research question related to possible differential developmental relations in the two parenting dimensions and children’s prosocial behaviors, our results evidenced similarities across 11 cultural groups. The present findings are the first to highlight developmental changes in family-related behavior and expectations for family obligations and children’s prosocial behaviors in diverse cultural groups. Concerning our second research question related to the extent to which initial level and changes influence subsequent changes in parental warmth and family values and children’s prosocial behaviors in different cultural groups, we found similar predictive effects in most of the cultural groups, with some exceptions in Kenya, Thailand, and Jordan. Consistent with previous work (Carlo et al., 2010; Carlo et al., 2018; Padilla-Walker & Carlo, 2007; Padilla-Walker et al., 2012), higher parental warmth at ages 9 and 10 predicted steeper mean-level increases in child prosocial behavior in the subsequent transitions (from T1 to T2 and from T2 to T3), meaning that the influence of sensitive, caring parenting remains important during adolescent transitions. In Jordan, Kenya, and Thailand the predictive associations between maternal warmth and child prosocial behaviors were only statistically significant later in pre-adolescence, which may indicate that warm parents in Jordan, Kenya, and Thailand differently contribute to the enactment of their children’s prosocial behaviors across adolescence. Future studies are needed to replicate these time-sensitive developmental effects.

The degrees of influence on different (although close) developmental periods for Kenyan, Thai, and Jordanian parents may underlie other cultural values and childrearing conceptions not considered in this study. All three countries share cultural values related to obedience and conformity to parental authority (see Sorbring & Lansford, 2019), and other parental educational practices that are more control-oriented could become salient for the study of child prosocial behaviors (Hoffman, 2000). In addition, in Kenya, Thailand, and Jordan the connection of parental warmth to prosocial behavior in late childhood could become evident in specific types of prosocial behaviors more tightly connected to the family context in late childhood (Padilla-Walker & Christensen, 2010), evidencing different cultural paths for prosocial development.

In summary, parental warmth and expectations of respect and care within the family and child prosocial behavior develop together. Several factors are likely underlying causes of this shared development (e.g., parents’ and children’s emotion regulation skills, children’s temperamental characteristics such as effortful control, parent-child attachment, open and supportive communication processes at home), and future studies are needed to explore whether the positive joint development among parental warmth, values, and prosocial behaviors still exists when these variables are controlled.

### Limitations, Future Directions, and Conclusions

Despite a number of important strengths (i.e., a multi-informant approach, a large cross-cultural sample of mothers, fathers, and their children followed longitudinally over 3 years), several limitations should be acknowledged. First, although our data were longitudinal, their correlational nature prevents us from establishing firm causal conclusions. Second, although we used a methodological approach that allowed us to consider both intra-individual and interindividual differences, we recognize that effects may have been attenuated by the presence of measurement error in the scales. Future studies using latent variable approaches are encouraged to obtain estimates of simultaneous development among parental warmth and values and children’s prosocial behaviors. Third, we used a measure of the tendency to be prosocial across contexts. Future research should introduce measures assessing prosocial tendencies toward different targets (i.e., family members, peers, strangers) to understand if the promotion of family obligations in adolescence fosters prosocial behaviors mainly toward family members or extends to other interpersonal contexts (e.g., toward peers). Fourth, future studies should include observational measures of prosocial behavior to have a stronger multi-method assessment of children’s prosocial actions. Fifth, we recognize that the reliability of prosocial behavior was lower than would be ideal. Although this was likely due to the availability of only three items for each informant (Cortina, 1993), we recommend that future studies rely on a measure with more indicators to obtain a fuller picture of children’s prosocial actions. Sixth, although we used a multi-informant approach, we acknowledge that the variability in the cross-informant correlations could indicate important unique perspectives in each rater’s point of view (De Los Reyes & Kazdin, 2005). Future studies should explore whether possible disagreements among informants (e.g., mother versus child) in parental warmth could signal later difficulties in children’s prosocial behavior. Seventh, we focused our analyses on the transition from late childhood to early adolescence. Later developmental periods also should be considered to understand whether these developmental processes in different cultures vary with time of life.

Family socialization variables are associated with children’s prosocial behaviors, an important component of positive youth development. Our findings provide evidence that caring relationships within the family are primary resources that set the stage for positive youth development. Behaving prosocially may pave the way for young people to thrive throughout development (Damon, 2004). In addition, enacting helping and caring behaviors within the family context may enhance children’s development of responsibility for themselves and the welfare of others. Focusing on the promotion of prosocial behaviors not only has the potential to support positive youth development, but also the potential to counteract or redirect negative trajectories of functioning.

## Supplementary Material

Supplementary Material
