# Supplementary material for: Positive Youth Development: Parental Warmth, Values, and Prosocial Behavior in 11 Cultural Groups
Source: J Youth Dev. Author manuscript; Available in PMC 2022 Jan 1. (PMC8651236; doi:10.5195/jyd.2021.1026)
Supplement: Supplementary Material [file NIHMS1754998-supplement-Supplementary_Material.pdf]

Positive Youth Development: Parental Warmth, Values, and Prosocial Behavior in 11 Cultural Groups

Supplementary Materials

Table S1. Sample Sociodemographic Characteristics by Country

	Colombia	Naples-Italy	Rome-Italy	Jordan	Kenya	Philippines	Sweden	Thailand	US- AA[a]	US-EA[a]	US-L[a]
					CHILD						
Age *M*(*SD*)	8.87(.59)	9.58(.55)	9.33(.87)	9.82(.57)	9.79(.93)	9.10(.45)	8.76(.43)	8.86(.64)	9.64(.64)	9.65(.59)	9.47(.73)
Gender (% male)	43.0%	48.5%	51.5%	52.6%	40.0%	50.5%	50.0%	50.0%	49.5%	60.0%	47.7%
Grade	3.69(.65)	4.24(.45)	3.80(.85)	4.01(.09)	3.80(.85)	3.26(.46)	2.67(.49)	3.41(.62)	3.98(.70)	4.12(.59)	3.78(.44)
N	101	99	101	114	95	107	97	116	91	100	85
					MOTHER						
Age	36.96 (7.67)	39.47 (5.38)	41.12 (5.18)	38.09 (5.92)	33.89 (6.11)	38.73 (6.39)	39.14 (4.83)	38.78 (6.17)	37.68 (8.40)	42.09 (6.47)	34.39 (6.05)
Education[b]	10.26 (5.62)	10.19 (4.30)	13.52 (4.09)	13.12 (2.26)	10.66 (3.64)	13.47 (3.80)	13.85 (2.45)	12.16 (4.55)	13.76 (2.15)	17.08 (2.95)	9.99 (4.27)
Employed	51.0%	42.4%	79.2%	25.4%	84.2%	67.3%	90.9%	87.1%	65.9%	73.7%	46.5%
N	101	99	101	113	95	107	99	116	91	100	85
					FATHER						
Age	40.21 (8.17)	42.02 (6.21)	44.24 (5.61)	43.18 (5.30)	40.51 (7.31)	40.80 (7.29)	41.86 (5.48)	40.86 (6.78)	39.71 (8.39)	43.56 (5.91)	37.24 (7.26)
Education[b]	10.01 (5.06)	10.94 (4.35)	13.26 (4.03)	13.62 (2.92)	12.50 (3.34)	13.34 (3.63)	13.98 (2.81)	12.90 (4.15)	13.42 (2.27)	17.25 (2.99)	10.36 (3.91)
Employed	89.9%	91.6%	97.9%	91.1%	93.5%	88.3%	94.0%	99.0%	82.2%	94.4%	84.5%
N	100	95	95	111	92	94	83	99	45	89	69
					FAMILY						
Married	68.3%	90.8%	77.0%	97.3%	95.8%	86.9%	57.3%	78.9%	36.3%	81.6%	55.3%
Nuclear [c]	70.0%	73.7%	83.2%	59.8%	47.4%	35.5%	98.0%	47.8%	91.2%	96.0%	68.6%
Dominant religion(s) (%)	Catholic (92.0)	Catholic (98.0)	Catholic (80.2)	Muslim (100.0)	Protestant (71.6)	Catholic (86.0)	None (51.0) Protestant (46.9)	Buddhist (99.1)	Protestant (75.3)	Protestant (57.6) Catholic (14.1)	Catholic (56.5) Protestant (28.2)
Median Income	5,000,000-10,000,000 Peso	11,000 – 15,000 Euro	30,000 – 40,000 Euro	3,500 – 7,000 Dinar	less than 400,000 Shilling	150,000 - 249,000 Peso	501,000-580,000 Krona	560,000 - 1,015,000 Baht	30,000 - 40,000 Dollar	61,000 – 70,000 Dollar	16,000 – 29,000 Dollar

Note. [a] AA = African American, EA = European American, L = Latino. [b] Years of education [c] Defined as 2 or fewer adults living in the household.

Table S2. Means and Standard Deviations for the Multi-Informant Constructs of Parental Warmth, Values, and Prosocial Behavior by Country

	Parental Warmth T1		Parental Warmth T2		Parental Warmth T3		Family Values T2		Family Values T3		Prosocial Behavior T1		Prosocial Behavior T2		Prosocial Behavior T3	
	Mean	*SD*	Mean	*SD*	Mean	*SD*	Mean	*SD*	Mean	*SD*	Mean	*SD*	Mean	*SD*	Mean	*SD*
Naples-Italy	3.68	0.24	3.67	0.22	3.63	0.29	4.08	0.35	3.99	0.36	3.76	0.60	3.78	0.60	3.64	0.63
Rome-Italy	3.63	0.20	3.59	0.25	3.50	0.31	3.90	0.29	3.89	0.32	3.73	0.56	3.71	0.52	3.64	0.59
Kenya	3.32	0.41	3.03	0.37	3.68	0.23	3.57	0.37	4.27	0.25	3.48	0.56	3.84	0.64	3.76	0.60
Philippines	3.63	0.27	3.62	0.27	3.57	0.27	4.07	0.33	4.07	0.35	3.54	0.58	3.47	0.49	3.59	0.54
Thailand	3.38	0.38	3.39	0.36	3.30	0.41	4.04	0.29	4.00	0.38	3.49	0.48	3.42	0.54	3.43	0.57
Sweden	3.77	0.15	3.80	0.17	3.75	0.19	3.30	0.31	3.25	0.33	3.71	0.48	3.78	0.49	3.53	0.50
US-AA	3.76	0.23	3.81	0.16	3.69	0.29	3.99	0.32	3.91	0.32	3.73	0.75	3.66	0.63	3.55	0.87
US-EA	3.80	0.19	3.85	0.13	3.78	0.19	3.63	0.32	3.56	0.34	3.68	0.52	3.72	0.53	3.54	0.65
US-L	3.78	0.19	3.72	0.28	3.67	0.32	4.18	0.30	4.19	0.35	3.86	0.60	3.85	0.62	3.78	0.79
Colombia	3.71	0.30	3.78	0.22	3.67	0.32	4.36	0.24	4.30	0.31	3.94	0.55	3.99	0.57	3.97	0.54
Jordan	3.52	0.35	3.54	0.31	3.40	0.40	4.25	0.34	4.18	0.40	3.59	0.64	3.51	0.69	3.55	0.73

Note. T1 (Time 1 = 9 years of age), T2 (Time 2 = 10 years of age) and T3 (Time 3 = 12 years of age). AA = African American, EA = European American, L = Latino.

Developmental trajectories of parental warmth, family values, and prosocial behaviors

In terms of developmental trajectories, multilevel models indicated a lack of mean-level changes in parental warmth in Naples (*b* = -.019, *p* = .090), Colombia (*b* = -.012, *p* = .269), Philippines, (*b* = -.013, *p* = .302), Sweden (*b* = -.006, *p* = .401), Thailand (*b* = -.031, *p* = .098), and U.S. European Americans (*b* = -.015, *p* = .077), whereas there was a slight decline in Rome (*b* = -.052, *p* < .001), U.S. African Americans (*b* = -.030, *p* = .038) and Latinos (*b* = -.031, *p* = .047), and Jordan (*b* = -.037, *p* = .005). Kenya showed a significant positive quadratic trend in parental warmth (*b* = .078, *p* = .008).

Family values did not change from T2 to T3 in Rome (*b* = -.023, *p* = .477), Jordan (*b* = -.063, *p* = .109), Philippines (*b* = -.004, *p* = .908), Sweden (*b* = -.027, *p* = .530), Thailand (*b* = -.067, *p* = .102), U.S. African Americans (*b* = -.074, *p* = .152), or Latinos (*b* = .021, *p* = .650), whereas they declined in Naples (*b* = -.102, *p* = .009), Colombia (*b* = -.066, *p* = .019), and U.S. European Americans (*b* = -.095, *p* = .007). Kenya, instead, showed an increase in family values from T2 to T3 (*b* = .707, *p* < .001).

Finally, no significant mean-level changes were found in Colombia (*b* = .007, *p* = .767), Rome (*b* = -.048, *p* = .107), Jordan (*b* = -.015, *p* = .558), Philippines (*b* = .022, *p* = .258), Thailand (*b* = -.018, *p* = .444), U.S. African Americans (*b* = -.069, *p* = .075), or Latinos (*b* = -.020, *p* = .605), whereas small significant declines were found in Naples (*b* = -.048, *p* = .003), Sweden (*b* = -.051, *p* = .020), and U.S. European Americans (*b* = -.049, *p* = .041). Kenya showed an increase in prosocial behavior over time (*b* = .075, *p* = .007).

Cross-Cultural Measurement Invariance

Before testing the relations among parental warmth, family values, and children's prosocial behaviors, we checked the invariance of the measures across the 11 cultural sites using the multiple-group factor analysis alignment method (Asparouhov & Muthén, 2014; Muthén & Asparouhov, 2014). The alignment method has been favored over the classic measurement invariance procedure in the presence of many groups given its advantage to estimate factor means and variances under the assumption of *approximate* rather than exact measurement invariance of factor loadings and intercepts (Asparouhov & Muthén, 2014). Overall, since fewer than 25% of the estimated parameters were non-invariant (Muthén & Asparouhov, 2014), we concluded that there was approximate measurement invariance in our cross-cultural study.[1]

Warmth

For mother-reported warmth, we found that only two intercepts were non-invariant (i.e., the intercept of the fourth parcel in Colombia at age 9 and the intercept of the second parcel in Rome, Italy, at age 12). For children's reports of maternal warmth, we found non-invariant intercepts in Thailand (the intercepts of the second and first parcel at 9 and 10 years of age, respectively), Sweden (the intercept of the third parcel at age 9), and Naples, Italy (the intercept of the second parcel at age 12). For children's reports of paternal warmth, we found that only the intercept of the second parcel at age 9 was non-invariant in Naples, Italy). Father-reported warmth did not show the presence of factor loadings and intercepts that were non-invariant across countries.

---

[1] To further reduce the complexity of the models and the number of parameters to be estimated, we modeled the constructs of warmth and family values (within each informant) as latent variables each measured by four parcels. Prosocial behavior, instead, was modeled using the three available items tapping into emotional support (Item 1 = "How often does your child try to make sad people happier?"), helping (Item 2 = "How often does your child help others with their homework?"), and sharing (Item 3 = "How often does your child let others use his/her toys?").

Values

For mother-reported family values, we found non-invariant factor loadings (at age 9) of the third and fourth parcels in three sites: the Philippines, Thailand, and the U.S. European Americans. At the intercept-level, there were non-invariant intercepts in Sweden (the intercepts of the second, third, and fourth parcel at both age 10 and 12), Kenya (the intercept of the second parcel at both age 10 and 12), Naples (Italy) and Jordan (the intercept of the first parcel at age 10), the Philippines (the intercept of the second parcel at age 12), and Colombia (the intercept of the third parcel at age 12). Father-reported family values showed a non-invariant factor loading for the third parcel in Thailand (at age 10) as well as at age 12 in Kenya, the Philippines, the U.S. African Americans, and Colombia. At the intercept-level, there were non-invariant intercepts in Sweden (the intercepts of the second, third, and fourth parcel at both age 10 and 12), Naples, Italy (the intercept of the first parcel at age 10), and Jordan (the intercept for the first parcel at age 12). For child-reported family values, we found only a non-invariant factor loading in Sweden for the third parcel at age 10. At the intercept-level, we found non-invariant intercepts in Sweden (the intercepts of the first and third parcel at age 10, and the intercept of the fourth parcel at both age 10 and 12), the Philippines (the intercept of the third parcel at age 10), Kenya (the intercept of the second parcel at age 12), Rome, Italy (the intercept of the fourth parcel at age 12).

Prosocial Behavior

For mother-reported children’s prosocial behavior, we found two non-invariant factor loadings in Colombia for the second and the third item at age 12, as well as non-invariant intercepts in Sweden (the intercepts of the second item at age 9, 10, and 12, and the intercept of the third item at age 9). For father-reported children’s prosocial behavior, we found non-

invariant intercepts in Kenya (the intercept of the second item at age 9), Sweden (the intercept of the third item at age 9, and the intercept of the second item at age 10 and 12), the U.S. African Americans (the intercept of the second item at age 10), and Colombia (the intercept of the third item at age 12)

Table S3. Parameter Estimates for the Final Multivariate Change Model (Model 3)

	Naples, Italy			Rome, Italy			Kenya			Philippines		
	b (β)	*SE*	*p*-value	*b* (β)	*SE*	*p*-value	*b* (β)	*SE*	*p*-value	*b* (β)	*SE*	*p*-value
Predictive effects												
b1. WaT1 → ΔWA[1]	-.424 (-.513)	.032	<.001	-.424 (-.388)	.032	<.001	-1.03 (-.829)	.108	<.001	-.424 (-.480)	.032	<.001
b2. WaT1 → ValT2	.281 (.208)	.049	<.001	.281 (.217)	.049	<.001	-.050 (-.055)	.114	.664	.281 (.238)	.049	<.001
b3. WaT1 → ΔPRO[1]	.387 (.189)	.079	<.001	.387 (.178)	.079	<.001	.141 (.078)	.178	.429	.387 (.232)	.079	<.001
b4. ProT1 → ΔWA[1]	.024 (.069)	.013	.071	.024 (.057)	.013	.071	.024 (.026)	.013	.071	.024 (.059)	.013	.071
b5. ProT1 → ValT2	.080 (.145)	.019	<.001	.080 (.165)	.019	<.001	.080 (.122)	.019	<.001	.080 (.150)	.019	<.001
b6. ProT1 → ΔPRO[1]	-.432 (-.513)	.031	<.001	-.432 (-.527)	.031	<.001	-.731 (-.563)	.120	<.001	-.432 (-.571)	.031	<.001
b7. WaT2 → ΔWA[2]	-.395 (-.346)	.056	<.001	-.395 (-.320)	.056	<.001	-.395 (-.374)	.056	<.001	-.395 (-.428)	.056	<.001
b8. WaT2 → ΔVAL[2]	.040 (.028)	.055	.468	.040 (.036)	.055	.468	.040 (.033)	.055	.468	.040 (.039)	.055	.468
b9. WaT2 → ΔPRO[2]	.222 (.089)	.099	.025	.222 (.119)	.099	.025	.222 (.107)	.099	.025	.222 (.133)	.099	.025
b10. ValT2 → ΔWA[2]	.083 (.106)	.036	.021	.083 (.074)	.036	.021	-.075 (-.071)	.078	.342	.083 (.108)	.036	.021
b11. ValT2 → ΔVAL[2]	-.347 (-.358)	.045	<.001	-.347 (-.346)	.045	<.001	-1.41 (-.855)	.078	<.001	-.347 (-.405)	.045	<.001
b12. ValT2 → ΔPRO[2]	.019 (.011)	.066	.771	.019 (.011)	.066	.771	.019 (.009)	.066	.771	.019 (.014)	.066	.771
b13. ProT2 → ΔWA[2]	.036 (.079)	.020	.075	.036 (.062)	.020	.075	.036 (.058)	.020	.075	.036 (.077)	.020	.075
b14. ProT2 → ΔVAL[2]	.032 (.058)	.022	.146	.032 (.063)	.022	.146	.032 (.045)	.022	.146	.032 (.062)	.022	.146

b15. ProT2 → ΔPRO[2]	-.297 (-.301)	.047	<.001	-.297 (-.342)	.047	<.001	-.585 (-.486)	.113	<.001	-.297 (-.357)	.047	<.001
d1. ΔWA[1] → ΔVAL[2]	-.023 (-.015)	.050	.637	-.023 (-.020)	.050	.637	-.023 (-.026)	.050	.637	-.023 (-.021)	.050	.637
d2. ΔWA[1] → ΔPRO[2]	-.092 (-.033)	.096	.340	-.092 (-.046)	.096	.340	-.092 (-.061)	.096	.340	-.092 (-.050)	.096	.340
d3. ΔPRO[1] → ΔWA[2]	-.030 (-.059)	.020	.135	-.030 (-.046)	.020	.135	-.030 (-.057)	.020	.135	-.030 (-.056)	.020	.135
d4. ΔPRO[1] →ΔVAL[2]	-.039 (-.061)	.024	.099	-.039 (-.066)	.024	.099	-.039 (-.063)	.024	.099	-.039 (-.064)	.024	.099
d5. ΔWA[1] → ΔWA[2]	-.192 (-.150)	.054	<.001	-.192 (-.144)	.054	<.001	-.346 (-.451)	.064	<.001	-.192 (-.188)	.054	<.001
d6. ΔPRO[1] →ΔPRO[2]	-.273 (-.241)	.048	<.001	-.273 (-.273)	.048	<.001	-.273 (-.261)	.048	<.001	-.273 (-.279)	.048	<.001

	cov (*r*)	*SE*	*p*-value	cov (r)	*SE*	*p*-value	cov (*r*)	*SE*	*p*-value	cov (*r*)	*SE*	*p*-value
Covariation												
c0. WaT1 ↔ ProT1	.027 (.206)	.005	<.001	.027 (.229)	.005	<.001	.027 (.122)	.005	<.001	.027 (.174)	.005	<.001
c1. ΔWA[1] ↔ ValT2	.008 (.159)	.002	<.001	.008 (.144)	.002	<.001	.045 (.368)	.015	<.001	.008 (.129)	.002	<.001
c2. ΔPRO[1] ↔ ValT2	.026 (.212)	.005	<.001	.026 (.257)	.005	<.001	.026 (.114)	.005	<.001	.026 (.243)	.005	<.001
c3. ΔPRO[1] ↔ ΔWA[1]	.009 (.135)	.003	.002	.009 (.109)	.003	.002	.009 (.046)	.003	.002	.009 (.123)	.003	.002
c4. ΔWA[2] ↔ ΔVAL[2]	.018 (.272)	.003	<.001	.018 (.243)	.003	<.001	.018 (.334)	.003	<.001	.018 (.348)	.003	<.001
c5. ΔPRO[2] ↔ΔVAL[2]	.029 (.207)	.005	<.001	.029 (.277)	.005	<.001	.029 (.214)	.005	<.001	.029 (.306)	.005	<.001
c6. ΔPRO[2] ↔ ΔWA[2]	.026 (.234)	.005	<.001	.026 (.228)	.005	<.001	.026 (.201)	.005	<.001	.026 (.331)	.005	<.001

Continued

	Thailand			Sweden			US-AA			US-EA		
	b (β)	*SE*	*p*-value	*b* (β)	*SE*	*p*-value	*b* (β)	*SE*	*p*-value	*b* (β)	*SE*	*p*-value
Predictive effects												
b1. WaT1 → ΔWA[1]	-.424 (-.541)	.032	<.001	-.424 (-.473)	.032	<.001	-.799 (-.791)	.188	<.001	-.424 (-.618)	.032	<.001
b2. WaT1 → ValT2	-.057 (-.068)	.097	.558	.281 (.149)	.049	<.001	.281 (.168)	.049	<.001	.281 (.157)	.049	<.001
b3. WaT1 → ΔPRO[1]	-.157 (-.102)	.145	.280	.387 (.153)	.079	<.001	.387 (.139)	.079	<.001	.387 (.164)	.079	<.001
b4. ProT1 → ΔWA[1]	.024 (.039)	.013	.071	.024 (.076)	.013	.071	.024 (.072)	.013	.071	.024 (.093)	.013	.071
b5. ProT1 → ValT2	.080 (.125)	.019	<.001	.080 (.214)	.019	<.001	.080 (.163)	.019	<.001	.080 (.121)	.019	<.001
b6. ProT1 → ΔPRO[1]	-.432 (-.367)	.031	<.001	-.432 (-.495)	.031	<.001	-.432 (-.523)	.031	<.001	-.432 (-.494)	.031	<.001
b7. WaT2 → ΔWA[2]	-.395 (-.303)	.056	<.001	-.395 (-.363)	.056	<.001	-.395 (-.260)	.056	<.001	-.395 (-.321)	.056	<.001
b8. WaT2 → ΔVAL[2]	.040 (.037)	.055	.468	.040 (.022)	.055	.468	.040 (.022)	.055	.468	.040 (.020)	.055	.468
b9. WaT2 → ΔPRO[2]	.222 (.122)	.099	.025	.222 (.080)	.099	.025	.222 (.070)	.099	.025	.222 (.059)	.099	.025
b10. ValT2 → ΔWA[2]	.083 (.060)	.036	.021	.083 (.143)	.036	.021	.083 (.112)	.036	.021	.083 (.148)	.036	.021
b11. ValT2 → ΔVAL[2]	-.379 (-.335)	.130	.004	-.347 (-.362)	.045	<.001	-.501 (-.574)	.142	<.001	-.347 (-.388)	.045	<.001
b12. ValT2 → ΔPRO[2]	.019 (.010)	.066	.771	.019 (.013)	.066	.771	.019 (.013)	.066	.771	.019 (.011)	.066	.771
b13. ProT2 → ΔWA[2]	.036 (.050)	.020	.075	.036 (.096)	.020	.075	.036 (.091)	.020	.075	.036 (.097)	.020	.075
b14. ProT2 → ΔVAL[2]	.032 (.055)	.022	.146	.032 (.052)	.022	.146	.032 (.070)	.022	.146	.032 (.055)	.022	.146
b15. ProT2 → ΔPRO[2]	-.297 (-.299)	.047	<.001	-.297 (-.312)	.047	<.001	.045 (.055)	.100	.655	-.297 (-.264)	.047	<.001

d1. ΔWA[1] → ΔVAL[2]	-.023 (-.019)	.050	.637	-.023 (-.012)	.050	.637	-.023 (-.018)	.050	.637	-.023 (-.010)	.050	.637
d2. ΔWA[1] → ΔPRO[2]	-.092 (-.045)	.096	.340	-.092 (-.030)	.096	.340	-.092 (-.040)	.096	.340	-.092 (-.021)	.096	.340
d3. ΔPRO[1] → ΔWA[2]	-.030 (-.041)	.020	.135	-.030 (-.071)	.020	.135	-.030 (-.069)	.020	.135	-.030 (-.072)	.020	.135
d4. ΔPRO[1] → ΔVAL[2]	-.039 (-.063)	.024	.099	-.039 (-.055)	.024	.099	-.039 (-.075)	.024	.099	-.039 (-.058)	.024	.099
d5. ΔWA[1] → ΔWA[2]	-.192 (-.131)	.054	<.001	-.192 (-.158)	.054	<.001	-.192 (-.172)	.054	<.001	-.192 (-.132)	.054	<.001
d6. ΔPRO[1] → ΔPRO[2]	-.273 (-.264)	.048	<.001	-.273 (-.249)	.048	<.001	-.273 (-.298)	.048	<.001	-.273 (-.212)	.048	<.001
	cov (*r*)	*SE*	*p*-value	cov (*r*)	*SE*	*p*-value	cov (*r*)	*SE*	*p*-value	cov (*r*)	*SE*	*p*-value
Covariation												
c0. WaT1 ↔ ProT1	.027 (.166)	.005	<.001	.027 (.334)	.005	<.001	.027 (.187)	.005	<.001	.027 (.280)	.005	<.001
c1. ΔWA[1] ↔ ValT2	.008 (.117)	.002	<.001	.008 (.193)	.002	<.001	.008 (.148)	.002	<.001	.008 (.230)	.002	<.001
c2. ΔPRO[1] ↔ ValT2	.026 (.181)	.005	<.001	.026 (.226)	.005	<.001	.026 (.159)	.005	<.001	.026 (.200)	.005	<.001
c3. ΔPRO[1] ↔ ΔWA[1]	.009 (.078)	.003	.002	.009 (.183)	.003	.002	.009 (.116)	.003	.002	.009 (.225)	.003	.002
c4. ΔWA[2] ↔ ΔVAL[2]	.056 (.451)	.015	<.001	.018 (.381)	.003	<.001	.018 (.301)	.003	<.001	.018 (.353)	.003	<.001
c5. ΔPRO[2] ↔ ΔVAL[2]	.029 (.187)	.005	<.001	.029 (.245)	.005	<.001	.029 (.227)	.005	<.001	.029 (.192)	.005	<.001
c6. ΔPRO[2] ↔ ΔWA[2]	.026 (.139)	.005	<.001	.026 (.376)	.005	<.001	.026 (.214)	.005	<.001	.026 (.269)	.005	<.001

Continued

	US-L			Colombia			Jordan		
	b (β)	SE	*p*-value	*b* (β)	*SE*	*p*-value	*b* (β)	*SE*	*p*-value
Predictive effects									
b1. WaT1 → ΔWA[1]	-.424 (-.357)	.032	<.001	-.424 (-.577)	.032	<.001	-.424 (-.533)	.032	<.001
b2. WaT1 → ValT2	.281 (.195)	.049	<.001	.281 (.331)	.049	<.001	.281 (.285)	.049	<.001
b3. WaT1 → ΔPRO[1]	.387 (.142)	.079	<.001	.387 (.211)	.079	<.001	-.012 (-.007)	.158	.941
b4. ProT1 → ΔWA[1]	.024 (.064)	.013	.071	.024 (.061)	.013	.071	.024 (.054)	.013	.071
b5. ProT1 → ValT2	.080 (.179)	.019	<.001	.080 (.181)	.019	<.001	.080 (.148)	.019	<.001
b6. ProT1 → ΔPRO[1]	-.432 (-.509)	.031	<.001	-.432 (-.448)	.031	<.001	-.432 (-.447)	.031	<.001
b7. WaT2 → ΔWA[2]	-.395 (-.349)	.056	<.001	-.395 (-.345)	.056	<.001	-.395 (-.335)	.056	<.001
b8. WaT2 → ΔVAL[2]	.040 (.036)	.055	.468	.040 (.040)	.055	.468	.040 (.031)	.055	.468
b9. WaT2 → ΔPRO[2]	.222 (.082)	.099	.025	.222 (.089)	.099	.025	.222 (.085)	.099	.025
b10. ValT2 → ΔWA[2]	.083 (.083)	.036	.021	.083 (.072)	.036	.021	.083 (.078)	.036	.021
b11. ValT2 → ΔVAL[2]	-.347 (-.352)	.045	<.001	-.347 (-.346)	.045	<.001	-.546 (-.470)	.110	<.001
b12. ValT2 → ΔPRO[2]	.019 (.008)	.066	.771	.019 (.008)	.066	.771	.019 (.008)	.066	.771
b13. ProT2 → ΔWA[2]	.036 (.077)	.020	.075	.036 (.075)	.020	.075	.036 (.064)	.020	.075
b14. ProT2 → ΔVAL[2]	.032 (.070)	.022	.146	.032 (.078)	.022	.146	.032 (.053)	.022	.146
b15. ProT2 → ΔPRO[2]	-.297 (-.268)	.047	<.001	-.297 (-.288)	.047	<.001	-.559 (-.456)	.105	<.001

d1. ΔWA[1] → ΔVAL[2]	-.023 (-.020)	.050	.637	-.023 (-.020)	.050	.637	-.023 (-.016)	.050	.637
d2. ΔWA[1] → ΔPRO[2]	-.092 (-.032)	.096	.340	-.092 (-.032)	.096	.340	-.092 (-.032)	.096	.340
d3. ΔPRO[1] → ΔWA[2]	-.030 (-.058)	.020	.135	-.030 (-.058)	.020	.135	-.030 (-.051)	.020	.135
d4. ΔPRO[1] → ΔVAL[2]	-.039 (-.075)	.024	.099	-.039 (-.084)	.024	.099	-.039 (-.060)	.024	.099
d5. ΔWA[1] → ΔWA[2]	-.192 (-.159)	.054	<.001	-.192 (-.146)	.054	<.001	-.192 (-.145)	.054	<.001
d6. ΔPRO[1] → ΔPRO[2]	-.273 (-.217)	.048	<.001	-.273 (-.236)	.048	<.001	-.273 (-.206)	.048	<.001
	cov (*r*)	*SE*	*p*-value	cov (*r*)	*SE*	*p*-value	cov (*r*)	*SE*	*p*-value
Covariation									
c0. WaT1 ↔ ProT1	.027 (.217)	.005	<.001	.027 (.180)	.005	<.001	.027 (.126)	.005	<.001
c1. ΔWA[1] ↔ ValT2	.008 (.134)	.002	<.001	.008 (.222)	.002	<.001	.008 (.107)	.002	<.001
c2. ΔPRO[1] ↔ ValT2	.026 (.208)	.005	<.001	.026 (.272)	.005	<.001	.026 (.153)	.005	<.001
c3. ΔPRO[1] ↔ ΔWA[1]	.009 (.090)	.003	.002	.009 (.116)	.003	.002	.009 (.074)	.003	.002
c4. ΔWA[2] ↔ ΔVAL[2]	.018 (.266)	.003	<.001	.018 (.304)	.003	<.001	.066 (.559)	.024	.007
c5. ΔPRO[2] ↔ ΔVAL[2]	.029 (.175)	.005	<.001	.029 (.235)	.005	<.001	.101 (.442)	.051	<.046
c6. ΔPRO[2] ↔ ΔWA[2]	.026 (.164)	.005	<.001	.026 (.188)	.005	<.001	.096 (.440)	.033	.004

Note. Regression coefficients (→) (both unstandardized [b] and standardized [β]) and covariation coefficients (↔), (both unstandardized [cov] and standardized [*r*]) with their standard errors (*SE*) are reported. Please refer to Figure 1 in the paper to graphically see the label associated with each parameter (e.g., b1). WA = Warmth. VAL = Family values. PRO = Children’s prosocial behaviors. US-AA = U.S. African American. US-EA = U.S. European American. US-L = U.S. Latino.
